# Abnormal beta power is a hallmark of explicit movement control in functional movement disorders

**DOI:** 10.1212/WNL.0000000000004830

**Published:** 2018-01-16

**Authors:** Tiago Teodoro, Anne Marthe Meppelink, Simon Little, Robert Grant, Glenn Nielsen, Antonella Macerollo, Isabel Pareés, Mark J. Edwards

**Affiliations:** From St. George's (T.T., G.N., M.J.E.), University of London, and St. George's University Hospitals NHS Foundation Trust, London, UK; Instituto de Medicina Molecular (T.T.), Faculdade de Medicina, Universidade de Lisboa & Serviço de Neurologia, Hospital de Santa Maria, Portugal; Department of Neurology (A.M.M.), University Medical Center Groningen and University of Groningen, the Netherlands; Sobell Department of Motor Neuroscience and Movement Disorders (S.L., A.M.), University College of London, Institute of Neurology; Faculty of Health, Social Care and Education (R.G.), Kingston University and St. George's, University of London, UK; and Neurology Department (I.P.), Hospital Ruber Internacional, Madrid, Spain.

## Abstract

**Objective:**

To determine whether sensorimotor beta-frequency oscillatory power is raised during motor preparation in patients with functional movement disorders (FMD) and could therefore be a marker of abnormal “body-focused” attention.

**Methods:**

We analyzed motor performance and beta-frequency cortical oscillations during a precued choice reaction time (RT) task with varying cue validity (50% or 95% congruence between preparation and go cues). We compared 21 patients with FMD with 13 healthy controls (HCs).

**Results:**

In HCs, highly predictive cues were associated with faster RT and beta desynchronization in the contralateral hemisphere (contralateral slope −0.045 [95% confidence interval (CI) −0.057 to −0.033] vs ipsilateral −0.033 [95% CI −0.046 to −0.021], *p* < 0.001) and with a tendency for reaching lower contralateral end-of-preparation beta power (contralateral −0.482 [95% CI −0.827 to −0.137] vs ipsilateral −0.328 [95% CI −0.673 to 0.016], *p* = 0.069). In contrast, patients with FMD had no improvement in RTs with highly predictive cues and showed an impairment of beta desynchronization and lateralization before movement.

**Conclusions:**

Persistent beta synchronization during motor preparation could reflect abnormal explicit control of movement in FMD. Excessive attention to movement itself rather than the goal might maintain beta synchronization and impair performance.

Sprinters waiting for the starting pistol to fire represent a fairly extreme example of movement preplanning and its benefits: movement preparation typically results in faster reaction times (RTs). One experimental method for probing this effect is the Posner paradigm whereby preparatory cues are given before a cue to move. When the preparatory cue provides useful information about the nature of the movement that will be required, RTs are characteristically faster.

Patients with functional (psychogenic) movement disorders (FMD) break this rule of normal movement. We previously found, using a version of the Posner paradigm, that patients with FMD were unable to take advantage of highly predictable conditions to improve performance.^1^ This is in keeping with a key clinical characteristic of their abnormal movement: movement that is explicitly controlled, for example, being asked to make a specific movement as part of neurologic examination, is abnormal, but when the same movement occurs in an unattended fashion, it is normal. We have previously proposed that this is due to an excessive attentional focus on movement, specifically a misdirection of attention away from the goal of movement and toward the mechanics of moving, i.e., monitoring the current state of the limb to be moved.^1^

If this hypothesis is correct, then we should be able to detect neural correlates of this misdirected attention. One potentially relevant correlate is power in the beta band of the EEG. Beta power characteristically suppresses and lateralizes before voluntary movement, and these changes are faster and more prominent before movement that is highly predicted.^2,3^ Beta power has been proposed to constitute an index of motor attention, with lower power reflecting higher attention to upcoming movement.^4^

We hypothesized that, in a precued RT task, patients with FMD would not improve their RTs when the precue was predictive of the upcoming movement and that this impairment would be associated with persistent beta synchronization and a failure to lateralize beta suppression compared to healthy controls (HCs).

Patients with FMD who were recruited into this study were about to start physiotherapy-based treatment for their symptoms. Therefore, we also hypothesized that clinical improvement at follow-up would be associated with improved RT in the setting of predictive precues and that this would be reflected by normalization of beta power suppression and lateralization before movement.

## Methods

### Participants

Patients with FMD were recruited from the same pool of patients who were being enrolled in the randomized feasibility study comparing specialized with standard physiotherapy for FMD.^5^ These were patients ≥18 years of age and with a clinically established diagnosis of FMD according to the Gupta and Lang^6^ criteria. Detailed inclusion and exclusion criteria have been published elsewhere.^5^ An additional exclusion criterion for the current study was persistent severe head tremor. HCs were healthy individuals matched for age and sex.

Participants with FMD were tested twice: before starting treatment (baseline) and at least 2 weeks after completing treatment (follow-up). HCs were assessed once. Participants with FMD were, as part of the feasibility study, randomized to receive specialized or standard physiotherapy.^5^ Treatment strategies are characterized elsewhere.^5^

Demographic and clinical information was collected at baseline and follow-up. Clinical improvement at follow-up was assessed with the Physical Function domain of the Short Form-36 Health Survey (SF-36) (version 1).^5,7^

### Precued choice RT task with varying cue validity

**Figure 1:**
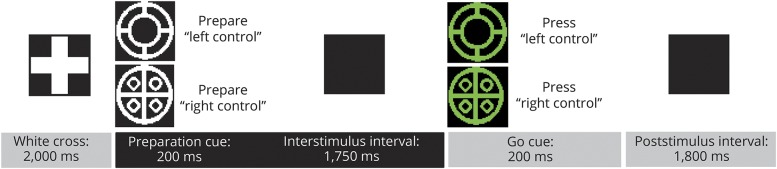
Trial structure Total duration is 5950 milliseconds. Preparation-to-move interval duration is 1,950 milliseconds, including preparation cue (200 milliseconds) and interstimulus interval (1,750 milliseconds). Participants were instructed to place the left index finger over the left Ctrl key and the right index finger over the right Ctrl key of a QWERTY computer keyboard.

Our behavioral experiment consisted of a Posner-type precued choice RT task with varying cue validity^1,8^ (figure 1). Before starting the formal paradigm, all participants completed a training block to ensure that they had optimal performance before proceeding to the main task.

Subsequently, there were 2 experimental conditions. In the highly predictable condition, preparation cues accurately predicted go cues in 95% of the trials (95% congruence). In the unpredictable condition, preparation cues accurately predicted go cues in only 50% of the trials (i.e., they had no predictive value).

The experiment was organized in 8 blocks of 50 trials each. Four blocks were highly predictable, and the other 4 were unpredictable. Block order was pseudo-randomized. Participants were instructed to press the key corresponding to the go cue as quickly as possible (either the left Ctrl key with left index finger or right Ctrl key with right index finger). They were also informed that in some blocks the preparation cues accurately predicted the go cues, while in the others they did not. They were not explicitly informed which type of block they were about to perform on each occasion. No feedback was given on accuracy or response time.

Response time in milliseconds (time from presentation of the go cue to key press) was calculated for each trial. Trials in which the preparation cue accurately predicted the go cue (congruent) were separated from those in which the prediction was incorrect (incongruent). Response times were separately averaged across trials for congruent and incongruent trials in each of the 2 conditions (95% and 50% congruence).

### Recording beta-frequency oscillatory activity

Continuous EEG was recorded from Ag/AgCl surface electrodes with a 32-channel ANT-EEG system conforming to the 5% electrode system. Electrode impedances were <5 kΩ. The sampling rate was 2,048 Hz.

Our reference was an average of all electrodes. All recordings were visually inspected for artifacts, and trials with prominent artifacts were discarded. We restricted our analysis to correct trials only and discarded trials in which participants pressed the wrong key or did not press any key (henceforth failed trials).

### Preprocessing

Statistical Parametric Mapping (12b, Wellcome Trust Centre for Neuroimaging, University College London, UK) and MATLAB (MathWorks, Natick, MA) were used for data processing. First, data were down-sampled from 2,048 to 250 Hz. It was then epoched to frames from −1 to 4 seconds relative to the onset of the preparation cue. Time frequency decomposition was performed with the Multitaper method.^9^ We averaged power over frequencies in the beta range (13–33 Hz). Data were rescaled by calculating the log ratio of the beta power relative to a baseline period ranging from −1 to 0 seconds relative to the onset of preparation cue.

Preprocessing resulted in baseline corrected beta power as a function of time for 4 different conditions: (1) 95% trial, right key press; (2) 95% trial, left press; (3) 50% trial, right press; and (4) 50% trial, left press.

For each of those conditions, we averaged the data from the FC1 and C3 electrodes (left hemisphere) and from the FC2 and C4 electrodes (right hemisphere), which are thought to record oscillatory activity from the sensorimotor cortex.

We next collapsed across the left/right distinction by averaging contralateral and ipsilateral beta power for each predictability setting (95% or 50%): left hemisphere activity during a right key press and right hemisphere activity during left key press, “contralateral” activity; and left hemisphere activity during a left key press and right hemisphere activity during right key press, “ipsilateral” activity.

The result was 4 datasets of beta power for each participant: (1) 95% condition, contralateral; (2) 95% condition, ipsilateral; (3) 50% condition, contralateral; and (4) 50% condition, ipsilateral.

Participants could preplan forthcoming the key press in the interval between the appearance of preparation and go cues. We focused on the period of maximum preparation by restricting our analysis to the second half of that interval (975 milliseconds preceding the go cue).

### Statistical analysis

Statistical analysis was performed with Stata (version 13.1, StataCorp, College Station, TX). Continuous variables were expressed as mean (SD) if normally distributed or median (interquartile range [IQR]) if not normally distributed. Categorical variables were expressed as frequencies and proportions.

The normality assumption was assessed by visual inspection of the distribution of the continuous variable and confirmed by Kolmogorov-Smirnov testing.

RTs were nonnormally distributed and were therefore transformed into their natural logarithm (Ln) to fulfill the normality assumption and thus be able to fit a multilevel mixed-effect linear model.

Our outcome measures were RT and beta power (see Preprocessing), including rate of beta desynchronization, representing the rate of beta power decrease in the second half of preparation to move, and end-of-preparation beta power, representing the level of beta power attained at the end of preparation to move (last 150 milliseconds before go cue).

Mixed-effects multilevel linear modeling allowed us to take into account the dependency in data caused by repeated measurements within participants for both RT and beta power. We fitted 3 models:

1. For RT, including the effects of group, predictability, and cue congruence, their interactions, and an individual-level random-effects factor. We performed 2 pairwise comparisons: Ln RT in congruent cue trials in 95% vs 50% conditions in both groups.
2. For rate of beta desynchronization, with the effects of group, predictability, laterality, and time, their interactions, and individual-level random-effects factors for intercept and slope.
3. For end-of-preparation beta power, with the effects of group, predictability, laterality, their interactions, and an individual-level random-effects factor.

For models 2 and 3, we performed 4 pairwise comparisons of contralateral and ipsilateral beta-band activity within 95% and 50% conditions for each group. Statistical significance was predefined as *p* < 0.05.

### Standard protocol approvals, registrations, and patient consents

The study was approved by the local ethics committee. Participants gave their informed written consent to take part in the studies.

## Results

### Clinical and demographic characteristics

We recruited 21 patients with FMD and 13 HCs, who were all assessed at baseline (table 1). Among patients with FMD, 11 were randomized to undergo specialized physiotherapy and another 12 to receive standard physiotherapy. They were then evaluated after a mean period of 4.7 (SD 1.7) weeks after treatment. Follow-up behavioral data (RT) from 1 patient with FMD were lost because of a technical problem on recording.^1^ At baseline, groups were well matched for age and sex. The proportion of left-handed participants was also similar between groups. After treatment, patients with FMD reported an increase in their SF-36 physical functional domain score (mean 30 [21.6 SD] at baseline vs 40 [30.0] at follow-up, *p* = 0.029).

**Table 1:**
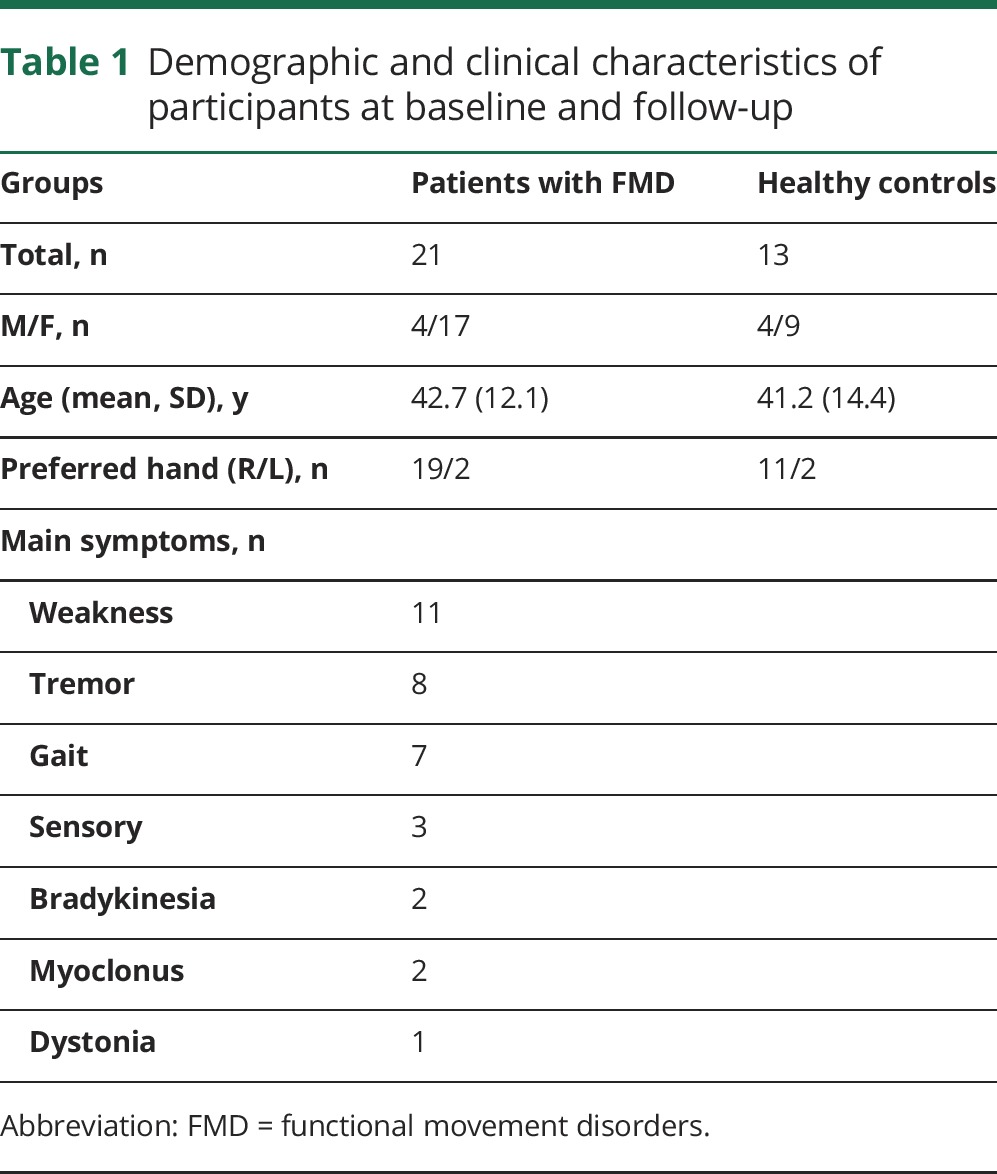
Demographic and clinical characteristics of participants at baseline and follow-up

### Main analysis: Patients with FMD at baseline vs HCs

HCs failed 1.3% (IQR 1.3%–2.3%) of trials, while patients with FMD failed 4.8% (IQR 1.5%–9.5%) (*p* = 0.022).

#### Behavioral effect of predictive and nonpredictive cues

**Figure 2:**
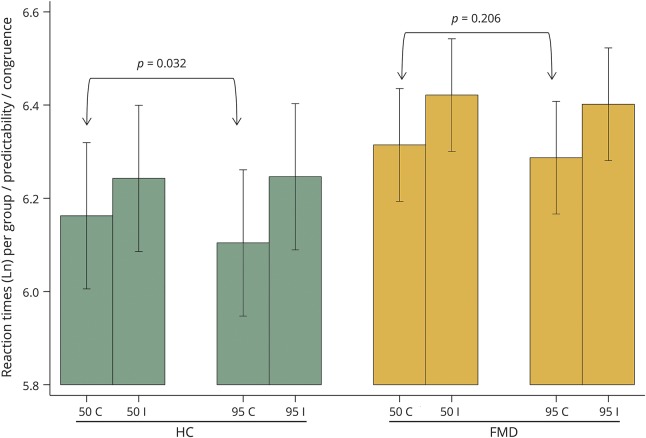
Natural logarithms of reaction times per group, predictability, and cue congruence Group: patients with functional movement disorders (FMD) vs healthy controls (HCs). Predictability: predictive (cuing blocks 95%) vs nonpredictive (cuing blocks 50%). Cue congruence: congruent (C) vs incongruent (I).

In HCs, response times for trials with predictive precues were faster than for nonpredictive precues [mean Ln(RT) difference −0.058, 95% confidence interval (CI) −0.112 to −0.005, *p* = 0.032] (figure 2 and table e-1, links.lww.com/WNL/A41). In contrast, there was no difference in response time in patients with FMD between predictive and nonpredictive precues [mean Ln(RT) difference −0.027, 95% CI −0.07 to 0.015, *p* = 0.206].

#### Beta desynchronization and end-of-preparation beta power with predictive and nonpredictive cues

**Figure 3:**
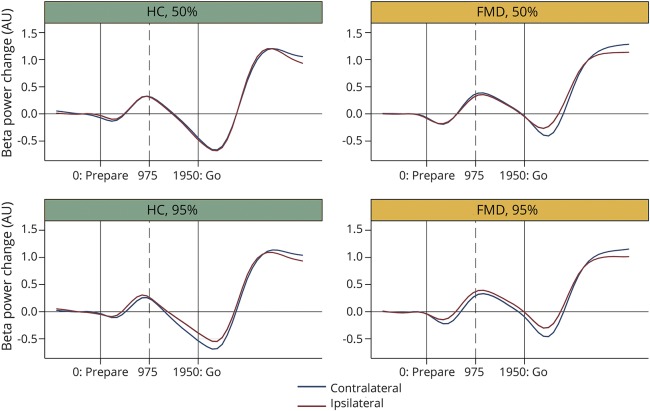
Baseline: FMD vs HCs Baseline-corrected beta power (log ratios) as a function of time. Blue trace shows contralateral hemisphere; red trace shows ipsilateral hemisphere. Beta power change (AU) was obtained by rescaling beta power, which consisted of calculating the log ratio of beta power relative to a baseline period ranging from −1 to 0 seconds relative to the onset of preparation cue. Beta slopes were obtained by kernel-weighted local polynomial regression of beta power change (AU) (yvar) on time (xvar). AU = arbitrary units; FMD = functional movement disorder; HC = healthy control; 50% = unpredictable blocks; 95% = highly predictable blocks; 0 (milliseconds) = prepare cue appearance; 975 (milliseconds) = halfway through the preparation-to-move period; 1,950 (milliseconds) = go cue appearance.

We compared, using mixed linear modeling, the rates of beta desynchronization before the go cue in the ipsilateral and contralateral hemispheres for blocks with predictive and nonpredictive precues in patients with FMD and HCs (figure 3 and table e-2, links.lww.com/WNL/A41). We found that only in HCs in blocks with predictive precues, there was a faster rate of beta desynchronization in the contralateral hemisphere (slopes: contralateral −0.045 [95% CI −0.057 to −0.033] vs ipsilateral −0.033 [95% CI −0.046 to −0.021], *p* < 0.001). In contrast, there was no difference in rate of beta desynchronization in HCs in blocks with nonpredictive cues (*p* = 0.664) and in patients with FMD with predictive (*p* = 0.801) or nonpredictive (*p* = 0.777) cues.

We also compared the baseline-corrected beta power at the end of preparation to move in the ipsilateral and contralateral hemisphere, for blocks with predictive and nonpredictive precues, in patients with FMD and HCs (figure 3 and table e-2, links.lww.com/WNL/A41).

Again, only in HCs and for blocks with predictive precues, there was a tendency for lower end-of-preparation beta power in the contralateral hemisphere (means: contralateral −0.482 [95% CI −0.827 to −0.137] vs ipsilateral −0.328 [95% CI −0.673 to 0.016], *p* = 0.069). In contrast, end-of-preparation beta power was similar in contralateral and ipsilateral hemispheres in HCs in blocks with nonpredictive cues (*p* = 0.602) and in patients with FMD with predictive (*p* = 0.300) or nonpredictive (*p* = 0.696) cues.

### Exploratory analysis: patients with FMD at follow-up vs baseline

Patients with FMD failed 4.8% (IQR 1.5%–9.5%) of trials at baseline and 2.9% (IQR 1.6%–10.8%) at follow-up (*p* = 0.117).

#### Behavioral effect of predictive and nonpredictive cues

Response times for trials with congruent cues were similar in blocks with predictive and nonpredictive precuing at both baseline (*p* = 0.430) and follow-up (*p* = 0.203) (table e-3, links.lww.com/WNL/A41).

#### Beta desynchronization and end-of-preparation beta power with predictive and nonpredictive cues

Contralateral and ipsilateral rates of beta desynchronization were similar at baseline in blocks with predictive (*p* = 0.874) and nonpredictive (*p* = 0.859) precueing and at follow-up for predictive (*p* = 0.302) and nonpredictive (*p* = 0.208) conditions (table e-4, links.lww.com/WNL/A41).

At follow-up after treatment, in blocks with predictive precues, there was a tendency for lower end-of-preparation beta power in the contralateral hemisphere (mean −0.068 [95% CI −0.313 to 0.176] vs 0.146 [95% CI −0.1 to 0.390], *p* = 0.065). In contrast, contralateral and ipsilateral beta powers were similar both at follow-up in blocks with nonpredictive cues (*p* = 0.717) and at baseline in predictive (*p* = 0.553) and nonpredictive (*p* = 0.823) conditions.

## Discussion

The behavioral data in this study replicate data from our previous study in people with FMD, showing that they appear unable to take advantage of precues that accurately predict the nature of an upcoming movement.^1^ They were different in this respect from HCs, who had faster response times when precues were informative.

Here, we demonstrate a neural correlate of this behavioral phenomenon. In HCs, beta power suppressed more deeply and lateralized more strongly during movement preparation in response to precues that were strongly predictive of the upcoming movement compared to when they were not. In contrast, people with FMD showed no such effect and had abnormally high and poorly lateralized beta power even in the setting of highly predictive precues.

We have previously proposed a key role for self-directed attention in the pathophysiology of FMD. Misdirected attention toward the body could, we have argued, both directly impair access to normal movement and activate abnormally strong top-down predictions related to abnormal movement.^1,10^ This could provide an explanation for the worsening of symptoms when attention can be focused on movement in contrast to improvement when movement happens in the setting of distraction.

Here, we have used an experimental paradigm that compares response times when movement can and cannot be prepared in advance of the cue to move. Healthy people are faster when they can prepare, but patients with FMD are not. We hypothesized that this effect relates to abnormal attentional focus onto the current sensorimotor state of the limb instead of focus toward the goal of upcoming movement. If this is correct, we would expect to see a failure of beta power suppression and lateralization in patients with FMD. This is indeed what we found in the patient group: their impaired task performance was associated with abnormally maintained beta power and failure of beta lateralization compared to HCs.

We suggest that abnormally maintained beta power in this experimental paradigm represents a state of abnormal attention toward the current sensorimotor state of the limb. However, we recognize that there are differences of opinion and much still to learn about the origins of changes in beta power before movement. There is a degree of consensus that beta power is at least related to the likelihood of a voluntary movement occurring or a change in the status quo.^11^ Current models of movement control suggest a system in which information regarding current sensory state and future movement plans is represented by probability distributions, the SD of which provides some measure of their relative strength or precision. Movement can be understood as a change in sensory state and occurs when future movement plans are stronger (more precise) than the current sensory state.^12^ We suggest that beta power is a candidate marker for precision in this context, and given how it decreases in preparation for movement, we suggest that it could index the strength (or precision) of the current sensory state of the body.

Our study is only the second to provide follow-up neurobiological data in functional neurologic disorders after treatment.^13^ Our exploratory comparison of patients with FMD before and after treatment did not confirm a behavioral improvement in the task (indexed by RTs) in spite of a significant increase in SF-36 scores indicating an overall improvement in symptoms. Nevertheless, at follow-up after treatment, we observed a tendency for recovery of lateralized beta suppression during motor preparation in the setting of predictive cuing conditions. Within the group, there was a lot of variability in response to physiotherapy treatment, and this may have obscured an effect on RT and beta desynchronization.

We acknowledge some limitations to our study. First, we analyzed averaged power over frequencies in the whole beta range from 13 to 33 Hz. This approach might have overshadowed any specific involvement of narrower ranges of frequencies within the beta band. Second, we analyzed data from the FC1 and C3 electrodes (left hemisphere) and FC2 and C4 electrodes (right hemisphere). Although these electrodes are thought to record oscillatory activity from the sensorimotor cortex, the spatial resolution of 32-channel EEG is known to be limited. This might also have contributed to between-participant heterogeneity in beta power measurements. Third, our sample size did not allow the performance of potentially interesting subgroup comparisons of patients with FMD at follow-up (e.g., responders vs nonresponders to treatment). Fourth, our analysis did not account for upper limb involvement by FMD, lateralized symptoms, or hyperkinetic vs hypokinetic phenomenologies. Fifth, our hypothesis is that abnormal beta power during movement preparation reflects excessive attention toward the body, but we accept that there are other possible causes. Finally, future studies could also examine other components of beta modulation related to movement, e.g., postmovement beta rebound.

Our data suggest that persistent beta synchronization and lack of lateralized beta desynchronization during motor preparation are signatures of abnormal explicit movement control in FMD. We propose that excessive self-directed attention, which is associated with an explicit mode of motor control, might interfere with beta desynchronization and through this impair motor performance.

## Glossary

CI: confidence interval
FMD: movement disorders
HC: healthy control
IQR: interquartile range
Ln: natural logarithm
RT: reaction time
SF-36: Short Form-36 Health Survey
